# Expression of RCK2 MAPKAP (MAPK-activated protein kinase) rescues yeast cells sensitivity to osmotic stress

**DOI:** 10.1186/s12934-015-0276-7

**Published:** 2015-06-12

**Authors:** V Kumar, A J Hart, T T Wimalasena, G A Tucker, D Greetham

**Affiliations:** School of Biosciences, University of Nottingham, Sutton Bonington Campus, Loughborough, Nottingham, LE12 5RD UK; Kingston Research Ltd, SaltEnd Chemicals Park, Hull, UK

## Abstract

**Background:**

*Saccharomyces cerevisiae* is the micro-organism of choice for the conversion of fermentable sugars during beverage or bioethanol fermentations. These fermentations are characterised by high osmotic stress on a yeast cell, with selected brewing fermentations beginning at 20–25% fermentable sugars and bioethanol fermentations at 13% fermentable sugars.

**Results:**

*RCK2* encodes for a MAPKAP (MAPK-activated protein kinase) enzyme and was identified on a locus by QTL analysis in yeast cells under osmotic stress, *RCK2* expression was placed under a tetracycline regulatable vector and rescued glucose, sorbitol or glycerol induced osmotic stress in an *rck2* null strain. A strain overexpressing *RCK2* had significantly faster fermentation rates when compared with the empty vector control strain.

**Conclusions:**

Presence of *RCK2* increased rates of glucose utilisation (~40 g glucose in first 8 h) during a 15% glucose fermentation and concurrent production of ethanol when compared with empty vector controls. Tolerance to osmotic stress using the tetracycline regulatable vectors could be turned off with the addition of tetracycline returning a *rck2* null strain back to osmotic sensitivity.

**Electronic supplementary material:**

The online version of this article (doi:10.1186/s12934-015-0276-7) contains supplementary material, which is available to authorized users.

## Background

Fermentations, whether for traditional beverage or bioethanol production, impose upon the microbe a variety of stresses. During industrial fermentation yeast strains are exposed to stresses such as oxygen concentration, osmotic pressure, pH, end-product (usually ethanol), nutrient availability and increasing temperature [1]. Osmotic stress can be defined as a situation where there is an imbalance in intracellular and extracellular osmolytes causing an alteration in cellular physiology [2]. In natural habitats, yeast are constantly exposed to fluctuations in osmotic stress which can lead to impaired functioning of the cell [3]. Within the brewing process osmotic stress is encountered upon pitching yeast cells into media (or wort) containing very high concentrations of dissolved fermentable sugars [1, 4]. Thus, resistance to osmotic stress is a desirable phenotypic attribute for improved yeast performance within a fermentation bioreactor.

Using F1 haploid segregants, from clean lineage *S. cerevisiae* strains, QTL on the yeast chromosome for several stress tolerances, including osmotic stress, were identified [5], genes within the loci have been assessed for their potential role in osmotic tolerance. *RCK2*, a MAPKAP (MAP activated protein kinase) was significantly up-regulated in yeast cells exposed to osmotic stress. Previous research has revealed that Rck2p is phosphorylated under oxidative or osmotic stress conditions, along with some other components within the high osmolarity glycerol (HOG) pathway [6]. Yeast responds to stress caused by an increase in osmolarity by activating the HOG MAPK cascade [7], and this cascade leads to an elevated synthesis of glycerol [8]. There are a great number of genes responsible for osmotic tolerance and the mechanism for gene regulation through the HOG pathway is still unclear. Around 50 genes are strongly dependant on *Hog1* [9], phosphorylation of Hog1p has been shown to influence the activity of metabolic enzymes [10]. Hog1p localization in the nucleus has been shown to be dependent on Rck2p activity [11]. Rck2p also acts on translation elongation factor 2 mediating a transient repression of protein synthesis [12] and regulates the translational expression of osmostress-regulated mRNA [13].

In this article, the importance of *RCK2* under osmotic stress was assessed, using phenotypic microarray assays along with performance in fermentation. *RCK2* expression was placed under a tetracycline regulatable vector in a Δ*rck2* null strain and tolerance to osmotic stress inducing chemicals such as d-glucose, sorbitol, glycerol and NaCl determined.

## Results

### Deletion of *RCK2* increases sensitivity to osmotic stress

**Figure 1:**
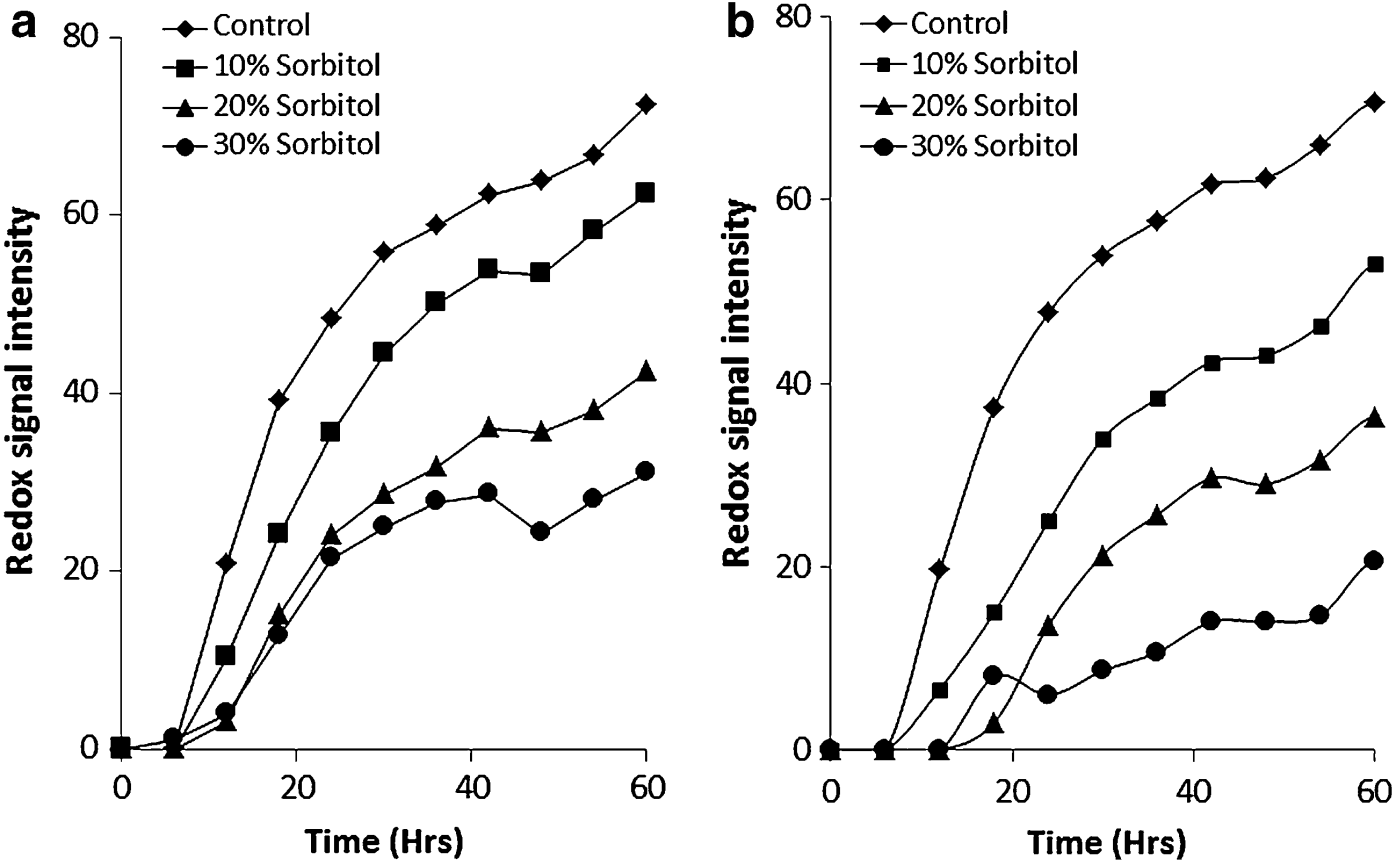
Phenotypic microarray analysis for *S. cerevisiae* BY4741 or Δ*rck2* under osmotic stress. **a** BY4741 under 0–30% sorbitol stress, **b** Δ*rck2* under 0–30% sorbitol stress. Mean ± SD (n = 3).

**Figure 2:**
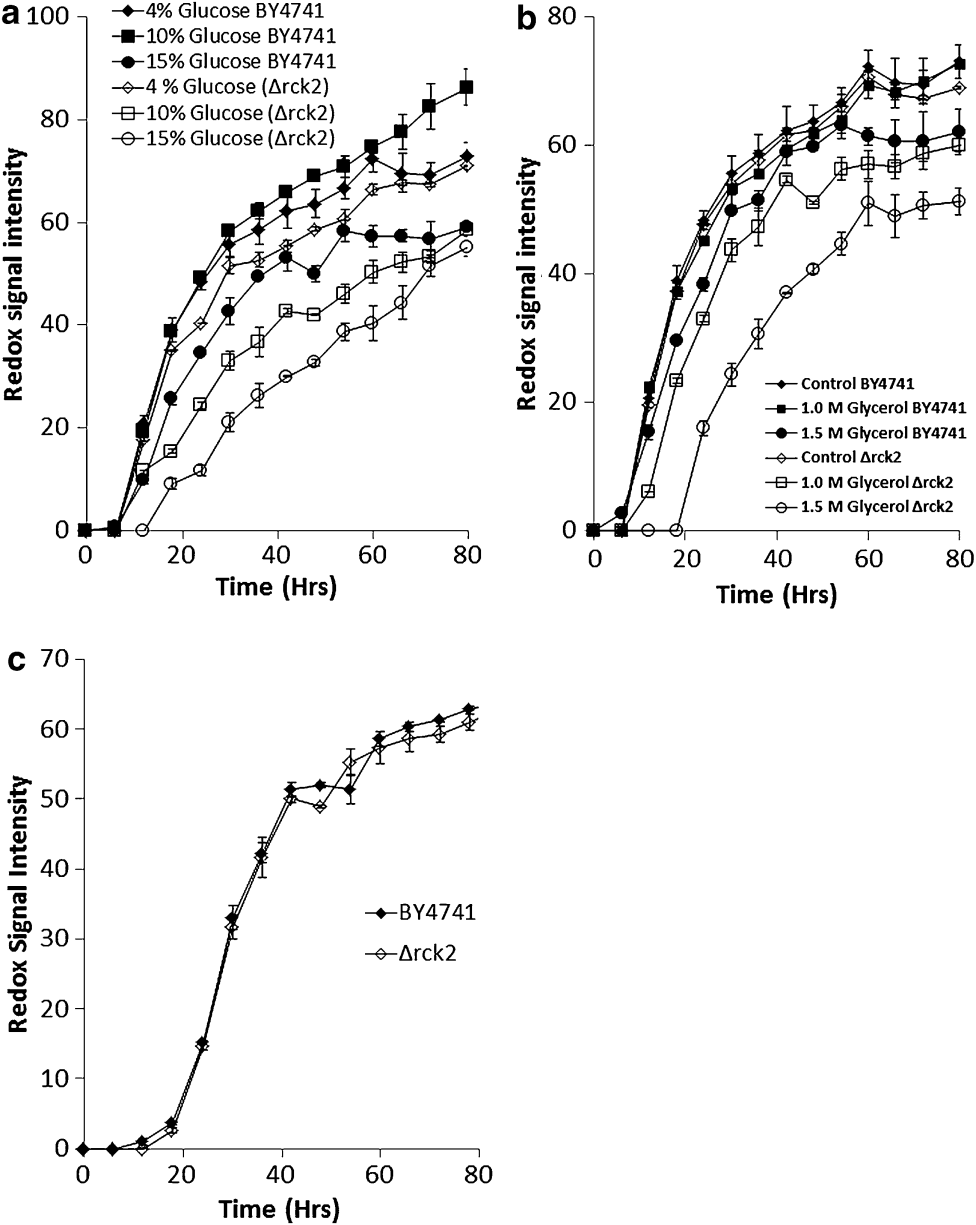
Phenotypic microarray analysis for *S. cerevisiae* BY4741 or Δ*rck2* under osmotic stress. **a** BY4741 or Δ*rck2* under 4,10, or 15% glucose stress, and **b** BY4741 or Δ*rck2* under control, 1.0 or 1.5 M glycerol stress. **c** BY4741 or Δ*rck2* under 2 M NaCl. Mean ± SD (n = 3).

The metabolic activity of wild type BY4741 and the Δ*rck2* strain during incubation in the presence of sorbitol (10–30%) was determined by use of a phenotypic microarray as measured by redox signal intensity (redox signal intensity has been defined previously [5]) (Figure 1a). It was observed that Δ*rck2* was more sensitive to the presence of sorbitol (10–30%) when compared with the background strain. In addition, Δ*rck2* also displayed increased sensitivity to the presence of increasing glucose and glycerol (Figure 2a, b); however, there was no difference between a Δ*rck2* strain and BY4741 in the presence of osmotic stress induced by the addition of NaCl (Figure 2c).

### Expression of *RCK2* in the Δ*rck2* strain recovers osmotic tolerance

**Figure 3:**
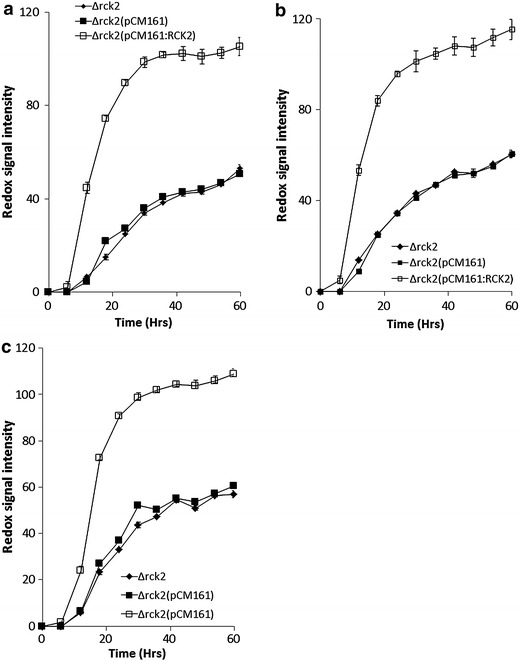
Phenotypic microarray analysis for *S. cerevisiae* Δ*rck2*, *S. cerevisiae* Δ*rck2*pCM161 and *S. cerevisiae* Δ*rck2*pCM161:*RCK2* under osmotic stress **a** 10% sorbitol stress, **b** 4% glucose stress, **c** 1.0 M glycerol stress. Mean ± SD (n = 3).

Insertion of a tetracycline regulatable vector (pCM161:*RCK2*) into a Δ*rck2* strain was assessed for impact on sensitivity to osmotic stress and compared with a strain carrying an empty vector (pCM161) as control. qPCR confirmed that expression of *RCK2* in the Δ*rck2*pCM161(*RCK2*), was 32-fold higher when compared with Δ*rck2*pCM161 (data not shown). Transformation with the pCM161 empty vector had no impact on metabolic output when under osmotic stress (10% sorbitol) compared with a Δ*rck2* strain (p = 0.9102), however, a Δ*rck2* strain containing a pCM161(*RCK2*) had significantly higher metabolic output (p = 0.0001) under this stress condition (Figure 3a). We also assessed for performance under increasing concentrations of glucose and observed that assays using a strain with an empty vector were identical to the Δ*rck2* strain, however, assays with a Δ*rck2* strain carrying pCM161(*RCK2*) revealed that there was a significant increase in metabolic output at all glucose concentrations (p = 0.0001) (Figure 3b), and metabolic output in the presence of 15% glucose was no different to that at 4% (p = 0.844) (Additional file 1: Figure S1). Assays using 20% glucose displayed a reduction in metabolic output in the Δ*rck2*pCM161(*RCK2*) strain compared with this strains output at 4–15% glucose (p = 0.0432), however, metabolic output was still significantly higher than a Δ*rck2*(pCM161) in the presence of 20% glucose (p = 0.0005) (Additional file 1: Figure S1).

### Confirmation of phenotypic microarray strain assessments using mini-fermentation analysis

**Figure 4:**
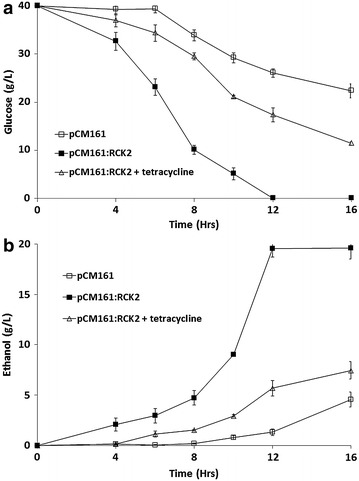
Performance of Δ*rck2*(pCM161) and Δ*rck2*(pCM161:*RCK2*) during a 40 g/L fermentation in the presence and absence of tetracycline **a** glucose utilisation **b** ethanol production. Mean + SD (n = 3).

The fermentation profiles of the strains using 40 g/L glucose were assessed in terms of glucose utilisation and ethanol production (Figure 4). It was observed that a strain with a pCM161(*RCK2*) vector utilised glucose and produced ethanol significantly faster than the empty vector control (p = 0.03) (Figure 4a, b). Addition of 1 µg/mL tetracycline reduced glucose utilisation and ethanol production when compared with absence of tetracycline (p = 0.03) (Figure 4a, b), however, glucose utilisation and ethanol production was significantly higher than in the pCM161 empty vector control (p = 0.03) (Figure 4a, b).

This data can be used to assess the efficiency of the conversion of glucose into ethanol. Under control conditions, the empty vector Δ*rck2* control strain had a 0.09 ± 0.003 g ethanol/g glucose conversion efficiency whilst the pCM161:*RCK2* strain had an efficiency of 0.48 ± 0.001 ethanol/g glucose conversion after 12 h. Addition of tetracycline reduced conversion efficiency to 0.24 ± 0.012. The theoretical maxima is 0.511 g ethanol per g of glucose consumed [14], therefore the pCM161:*RCK2* strain was converting glucose into ethanol at near theoretical maximum during a fermentation in the presence of 40 g/L glucose.

### Overexpression of *RCK2* improved fermentation in the presence of 15% glucose

**Figure 5:**
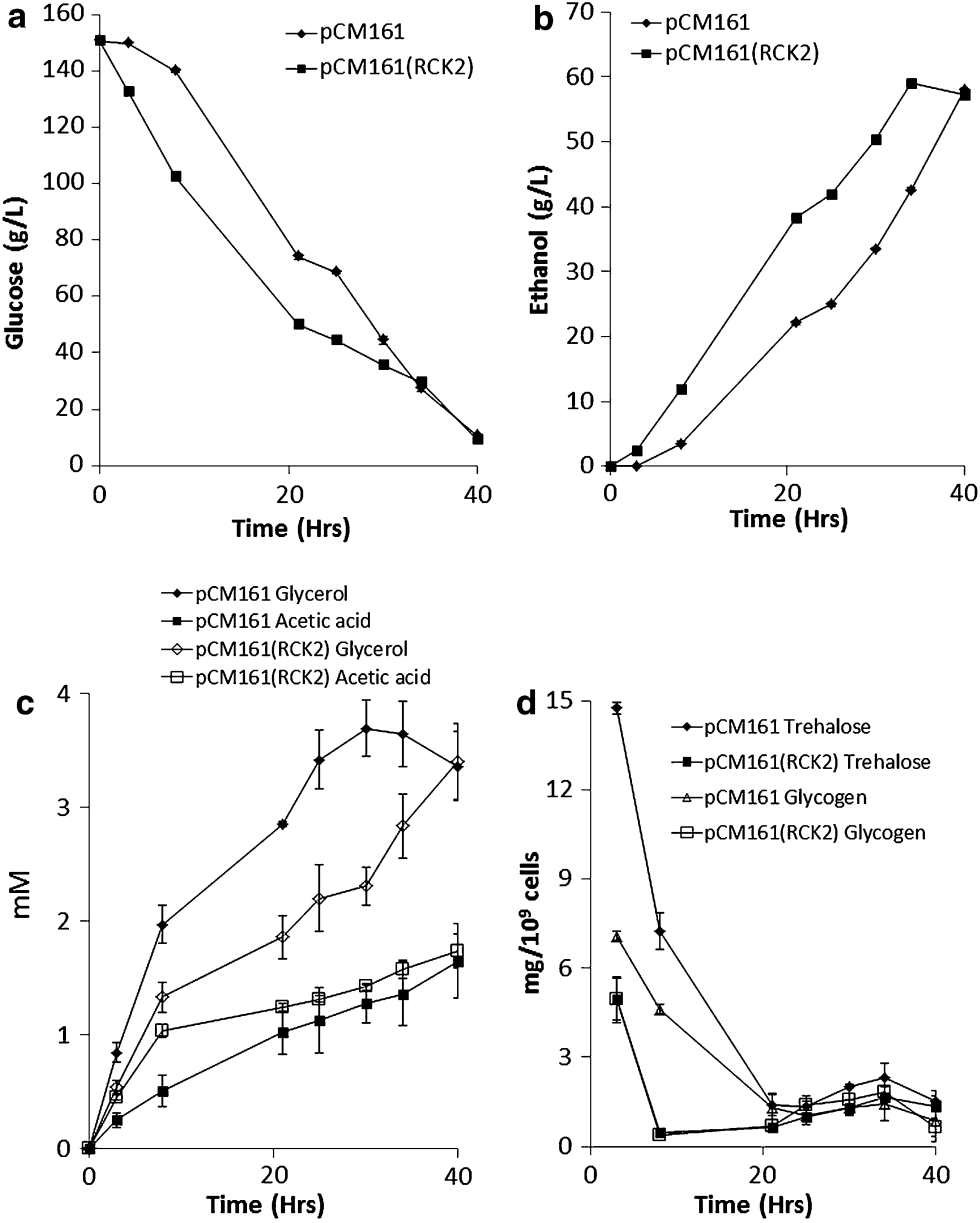
Performance of Δ*rck2*(pCM161) and Δ*rck2*(pCM161:*RCK2*) during a 150 g/L fermentation. **a** glucose utilisation **b** ethanol production **c** acetic acid (mM) and glycerol (mM) concentrations **d** trehalose and glycogen concentrations (mg/10^9^ cells). Mean + SD (n = 3).

Weight loss experiments using Δ*rck2*pCM161(*RCK2*) and Δ*rck2*pCM161 revealed that presence of *RCK2* significantly improved rates of fermentation in the presence of 15% glucose (Additional file 2: Figure S2). There have been a number of studies where ethanol fermentations by *S. cerevisiae* have been started with initial glucose concentration of around 100 g/L [15] so we tested these strains in 2 L fermentation in the presence of 150 g/L glucose and monitored glucose utilisation, ethanol production, and production of metabolites such as acetic acid and glycerol; we also determined the concentration of stress response storage carbohydrates in the cell such as trehalose and glycogen. Results revealed that presence of *RCK2* significantly speeded up utilisation of glucose and production of ethanol when compared with the empty vector controls (Figure 5a, b), fermentations containing *RCK2* also produced more acetic acid than the empty vector control (Figure 5c), however, concentrations of glycerol were significantly lower than with the empty vector control (Figure 5c).

An assessment of accumulation of storage carbohydrates such as trehalose and glycogen revealed that there was initially higher concentrations of these polymers in the empty vector controls when compared with the strain containing *RCK2* (Figure 5d). Trehalose and glycogen concentrations dropped rapidly at the start of the fermentation and after 20 h there was no difference between the yeast types (Figure 5d).

### Carbon partitioning during fermentations using Δ*rck2*pCM161 or Δ*rck2*pCM161(*RCK2*)

Carbon balance analysis was carried out for yeast strains Δ*rck2*pCM161 and Δ*rck2*pCM161(*RCK2*) during the 150 g/L glucose fermentations (Table 1). For carbon analysis, the cultivation period was divided into two phases, I (0–21 h) and II (21–40 h). The two strains differed in consumption of glucose and ethanol formation in each phase with Δ*rck2*pCM161 assimilating 429.1 mM and Δ*rck2*pCM161(*RCK2*) assimilating 559.4 mM glucose, respectively in phase I. The difference became more significant with respect to ethanol production, Δ*rck2*pCM161(*RCK2*) strain generated about 73% more ethanol (831.1 mM) in comparison with Δ*rck2*pCM161 (481.1 mM) in the first 21 h, this trend was reversed in the second phase of the fermentation (21–40 h) where consumption 354.1 and 228.2 mM glucose by Δ*rck2*pCM161 and Δ*rck2*pCM161(*RCK2*) resulted in 781.3 and 411.6 mM ethanol, respectively.

**Table 1:** Carbon balance for batch cultivation of Δ*rck2*pCM161(*RCK2*) and Δ*rck2*pCM161(*RCK2*) strains during a 15% glucose fermentation

Substrate and products	Phase I (0–21 h)			Phase II (21–40 h)		
	Amount (mM)	Carbon (mM)	Carbon (%)	Amount (mM)	Carbon (mM)	Carbon (%)
Δ*rck2*pCM161						
Glucose	426.1	2556.6	100.0	354.1	2124.6	100.0
Ethanol	481.1	962.2	37.6	781.3	1562.6	73.5
Glycerol	2.9	8.7	0.3	0.5	1.5	0.1
Acetic acid	1.0	2.0	0.1	0.6	1.2	0.1
Trehalose	1.2	7.2	0.3	0.0	0.0	0.0
Glycogen	1.1	6.6	0.3	0.4	2.4	0.1
CO_2_	481.1	481.1	18.8	781.3	781.3	36.8
Carbon recovery	57.6			110.6		
Δ*rck2*pCM161(*RCK2*)						
Glucose	559.4	3356.4	100.0	228.4	1370.4	100.0
Ethanol	831.1	1662.2	49.5	411.6	823.2	60.1
Glycerol	1.9	5.7	0.2	1.5	4.5	0.3
Acetic acid	1.2	2.4	0.1	0.5	1.0	0.1
Trehalose	1.0	6.0	0.2	0.4	2.4	0.2
Glycogen	1.1	6.6	0.2	0.4	2.4	0.2
CO_2_	831.1	831.1	24.8	411.6	411.6	30.0
Carbon recovery	74.9			90.9		

## Discussion

The conversion of sugars into ethanol is the prime function of yeast during a brewing or bioethanol fermentation, the osmotic effect of unfermented sugars and ethanol inhibition are major stresses imposed on the yeast cell which inevitably affect their growth and rates of fermentation [4, 16]. Adaptation to hyperosmotic stress via the high osmolarity glycerol (HOG) transduction pathway is well defined in yeast [17].

Rck2p is a serine/threonine protein kinase homologous to mammalian calmodulin kinases, which requires phosphorylation for activation, phosphorylation is transiently increased during osmotic stress or in strains overexpressing the HOG pathway [18]. A QTL analysis for yeast revealed that on chromosome XII there were genes responsible for osmotolerance, upon further examination of the QTL, *RCK2* was found to be up-regulated in yeast cells under osmotic stress [5, 19].

Placing *RCK2* expression under a tetracycline regulatable vector, we measured performance under osmotic stress and compared this with the performance of a Δ*rck2* strain. Performance of the Δ*rck2* strain under osmotic stress (glucose, sorbitol, or glycerol) confirmed that absence of *RCK2* was characterised by osmosensitivity. Expression of *RCK2* in a *rck2* null background conferred resistance to osmotic stress in terms of both increased metabolic output and rates of fermentation. Addition of tetracycline reduced *RCK2* expression and reduced the rates of fermentation.

Overexpression of *RCK2* has been observed previously to confer osmotic tolerance in *hog1* null yeast cells when induced by the addition of NaCl [6], tolerance as measured by inhibition was believed to be downstream of Hog1 phosphorylation. Rck2p along with other protein kinases forms a sub-family of MAPK enzymes which incorporates a characteristic glycine loop in their C-terminal domain [20]. In general, there is a down-regulation in translation in cells under osmotic stress, however, this down-regulation is mitigated in *hog1* or *rck2* mutants [18] and Rck2p has been shown to phosphorylate elongation factor 2 a component of translation regulation [12, 18] indicating an importance of Rck2p in maintaining translation under osmotic stress conditions.

## Conclusion

Results reported here are the first example of how overexpressing *RCK2* conferred resistance to osmotic stress when induced by the presence of reducing sugars during a fermentation, results revealed that the improved rates of fermentation in a cell overexpressing *RCK2* was not characterised by an increase in glycerol when compared with an empty vector control indicating that the role of Rck2p in osmotically stressed cells is not solely through an up-regulation of the HOG pathway which is characterised by an increased in glycerol production [8]. Future work will be to determine the cellular role of *RCK2* in osmotic stressed yeast cells and to track *RCK2* expression during fermentations.

## Experimental procedures

### Yeast strains and growth conditions

Yeast strains employed in this work derive from *S. cerevisiae* BY4741 (Table 2), all strains were grown in YPD [1% (w/v) yeast extract (Oxoid); 2% (w/v) Bacto-peptone (Oxoid); 2% (w/v) glucose]. Strains were deleted for tryptophan biosynthesis (*trp1*::URA3) using pAG60 (Euroscarf, Frankfurt, Germany) with a URA3 selectable marker using primers (Table 3). A Δ*rck2* null mutant was obtained from Euroscarf (Frankfurt, Germany) and *TRP1* was also deleted from this strain as above for BY4741.

**Table 2:** Strains used in this study, all strains are derived from BY4741 (MATa *his3*∆0 *leu2*∆0 *met15*∆0 *ura3*∆0), with the additional *TRP1* gene knocked out to allow for selection with pCM plasmids

Strains used in this study	Integrative plasmid^a^
BY4741Δ*trp1* (MATa *his3*∆0 *leu2*∆0 *met15*∆0 *ura3*∆0 *trp1*Δ0)	–
BY4741Δ*trp1Δrck2*	–
BY4741Δ*trp1*Δ*rck2*	pCM161
BY4741Δ*trp1*Δ*rck2*	pCM161 (*RCK2*)

^a^Integrative plasmids were constructed as indicated in “Plasmid construction”.

**Table 3:** Primers used in this study for the knockout of *TRP1* and insertion of pCM vectors into yeast strains

Primers	Sequence
*TRP1* knockout forward	cgccagatggcagtagtggaagatattctttattgaaaaatagcttgtcaATGACAGTCAACACTAAGACCTATA
*TRP1* knockout reverse	ttttatgcttgcttttcaaaaggcctgcaggcaagtgcacaaacaatactTTATAATTGGCCAGTCTTTTTC
pCM161 forward	ATCGATATGCTTAAAATAAAGGCC
pCM161 reverse	GGATCCCTATTCCCTGATAGTGGC

### Plasmid construction

Plasmid pCM161 is a centromeric yeast plasmid, marker *TRP1*, tetracycline repressed expression of lacZ under the control of the *tetO*_2_ promoter. For the construction of the pCM161(RCK2), a PCR product encoding for *RCK2* with relevant restriction enzyme sites appropriate for ligation into the vectors was prepared. For cloning into pCM161, *Cla1* and *BamH1* restriction enzymes (NEB) were used and the relevant digest site added to the forward and reverse primers (Table 3).

### PCR of RCK2 from genomic DNA

*S. cerevisiae* BY4741 was grown to stationary phase, cells harvested and broken with glass beads using a MagNalyser (Roche, Burges Hill, UK) bead beater for 30 s at 4°C, before incubating on ice for 15 min to precipitate proteins. Cell debris and proteins were harvested by centrifugation for 15 min (17,000×*g* at 4°C). The cell-free supernatant was used for the extraction of total DNA using an isolation kit from. PCR was performed using primers (pCM161F—ATCGATATGCTTAAAATAAAGGCC and pCM161R—GGATCCCTATTCCCTGATAGTGGC–GGATCCCTATTCCCTGATAGTGGC). PCR products using above primers were digested and linearized digested plasmids with appropriate restriction enzymes were ligated using the Quick ligation mix (New England Biosciences, US,) and the ligation mixture used in a standard lithium acetate transformation [21] and plated out on appropriate selection agar plates.

### qPCR

Strains BY4741, Δ*rck2* and Δ*rck2* containing plasmids were grown to the mid-logarithmic stage of growth in 4% SD-Trp media 30°C and stressed by the addition of 20% sorbitol for 15 min, rotated at 150 rpm. Cells were broken with glass beads using a MagNalyser (Roche, Burges Hill, UK) bead-beater for 30 s at 4°C, before incubating on ice for 15 min to precipitate proteins. Cell debris and proteins were harvested by centrifugation for 15 min (17,000×*g* at 4°C). The cell-free supernatant was used for the extraction of total RNA using an isolation kit from Qiagen (Hilden, Germany) and cDNA prepared using a first strand cDNA synthesis kit (GE Healthcare, Bucks, UK). Transcriptional levels were determined by qPCR using the following conditions follows: 0.5 ng/µl cDNA, 6.25 µM forward primer, 6.25 µM reverse primer, 5 µL of 2× SYBR Green master mix (Applied Bio Systems) and made up to 20 µL using molecular grade water.

All data was compared against *ACT1* which encodes for actin a structural protein in yeast as an internal normaliser and expression data from genes within the relevant loci were presented as fold-change in comparison to *ACT1* transcript levels in control and stress conditions.

### Phenotypic microarray analysis

For phenotypic microarray (PM) analysis, medium was prepared as described previously [5]. Osmotic stress was induced by addition of 10–30% sorbitol, 1–2 M glycerol, or 1–2 M NaCl using 80% sorbitol, 5 M glycerol or 10 M NaCl as stock solution as appropriate. Appropriate amounts from these stocks were added to the wells of the phenotypic microarray assay displacing water to maintain a final volume of 120 µL in the assay.

### Confirmation of phenotypic microarray results using mini fermentation vessels

Fermentations were conducted in 180 mL mini-fermentation vessels (FV). Cryopreserved yeast colonies were streaked onto YPD plates and incubated at 30°C for 48 h. Colonies of yeast strains were used to inoculate 20 mL of YPD broth and incubated in an orbital shaker at 30°C for 24 h. These were then transferred to 200 mL of YPD and grown for 48 h in a 500 mL conical flask shaking at 30°C. Cells were harvested and washed three times with sterile RO water and then re-suspended in 5 mL of sterile water. For control conditions, 1.5 × 10^7^ cells mL^−1^ were inoculated in 99.6 mL of medium containing 40 g/L (4%) glucose, 2% YNB-trp with 0.4 mL reverse osmosis (RO) water. For stress conditions, 1.5 × 10^7^ cells mL^−1^ were incubated in 99.6 mL of medium containing 40 g/L glucose, 2% YNB-trp with 75 mM acetic acid, Volumes of media were adjusted to account for the addition of the inhibitory compounds (~400 μL) to ensure that all fermentations began with the same carbon load. Fermentations with 80, 100 or 150 g/L glucose had a starting carbon load of 80, 100 or 150 g/L in a final volume of 100 mL with pitching levels identical to fermentations with 40 g/L glucose.

Anaerobic conditions were prepared using a sealed butyl plug (Fisher, Loughborough, UK) and aluminium caps (Fisher Scientific). A hypodermic needle attached with a Bunsen valve was purged through rubber septum to facilitate the release of CO_2_. All experiments were performed in triplicate and weight loss was measured at each time point. Mini-fermentations were conducted at 30°C, with orbital shaking at 200 rpm.

### 15% glucose fermentation in 2 L fermentation vessels

After growing the yeast strains in 180 mL mini-fermentation vessels, batch fermentations of two strains (pCM161 and pCM161(*RCK2*)) were carried out in 2 L vessels with a working volume of 1.5 L SD-trp medium. The initial glucose concentration was 150 g/L. The temperature and agitation was controlled to 30°C and 250 rpm, respectively. The stirring was achieved using magnetic beads. The pH was not controlled during the fermentation but initial pH was adjusted to pH 4.0–4.5 using phosphoric acid. These vessels have provisions for sparging of air and the experiments were conducted under microaerobic conditions which were achieved by sparging air at an aeration rate of 0.1 vvm.

### Detection of glucose ethanol, acetic acid and glycerol from FV experiments via HPLC

Glucose, ethanol, acetic acid and glycerol were quantified by HPLC. The HPLC system included a Jasco AS-2055 Intelligent auto sampler (Jasco, Tokyo, Japan) and a Jasco PU-1580 Intelligent pump (Jasco). The chromatographic separation was performed on a Rezex ROA H^+^ organic acid column, 5 μm, 7.8 mm × 300 mm, (Phenomenex, Macclesfield, UK) at ambient temperature. The mobile phase was 0.005 N H_2_SO_4_ with a flow rate of 0.5 mL/min. For detection a Jasco RI-2031 Intelligent refractive index detector (Jasco) was employed. Data acquisition was via the Azur software (version 4.6.0.0, Datalys, St Martin D’heres, France) and concentrations were determined by peak area comparison with injections of authentic standards. The injected volume was 10 μL and analysis was completed in 40 min. All chemicals used were analytical grade (>95% purity, Sigma-Aldrich, UK).

### Determination of intercellular glycogen and trehalose concentrations

Intracellular glycogen and trehalose were estimated based on the method of Parrou and Francois [22]. Frozen yeast samples were thawed on ice and an appropriate cell suspension volume containing 1 × 10^9^ cells was centrifuged at 3,000 rpm for 5 min at 4°C. The pellet was washed three times with distilled water. Cells were lysed by resuspension of the pellet in sodium carbonate (0.25 mL; 0.25 M) and were incubated at 95°C for 2 h followed by the addition of sodium acetate (0.6 mL; 0.2 M) and acetic acid (0.15 mL; 1 M). 0.5 mL aliquots were then assessed for glycogen and trehalose concentrations. Glycogen and trehalose were broken down into glucose by adding 10 μL of α-amyloglucosidase (10 mg/mL; 59.9 Units/mg; Fluka Biochemika, Steinheim) or 10 μL trehalase (3 m Units; Sigma-Aldrich, UK) respectively followed by incubation at 57 and 37°C for 14 h. Post incubation, samples were centrifuged (3,500 rpm; 5 min) and the supernatant (0.1 mL) containing liberated glucose was quantified using the Megazyme Glucose Assay kit (GOPOD, Megazyme, Ireland) at an optical density of 510 nm. Analysis for each time point was conducted in triplicates and results were expressed in concentration of glucose as a function of cell number.

### Carbon analysis

Carbon analysis was calculated based on the consumed substrate and accumulated metabolites, glucose, ethanol, glycerol, acetic acid, trehalose, glycogen and carbon dioxide [23]. The evolution of carbon dioxide was estimated using the following equation: C_6_H_12_O_6_ → 2C_2_H_5_OH + 2CO_2_ and CO_2_ coming from other sources such as the TCA cycle was not included into the calculations. The carbon used for biomass formation was not taken into account due to lack of information on biomass composition of yeast cells.

### Statistical analysis

Data derived from phenotypic microarrays was analysed for analysis of variance (ANOVA) using ezANOVA (http://www.cabiatl.com/mricro/ezanova), with statistical significance signified by use of, * = 0.05% significance, ** = 0.01% significance and *** 0.001% significance.

## Additional files

**Additional file 1: Figure S1.** Phenotypic microarray analysis for *S. cerevisiae* Δ*rck2*(pCM161:*RCK2*) under 4 and 15% glucose. Mean + SD (n = 3).
**Additional file 2: Figure S2.** Fermentation profiles for *S. cerevisiae* Δ*rck2* pCM161 or *S. cerevisiae* Δ*rck2* pCM161:*RCK2* under osmotic stress (A) performance of *S. cerevisiae* Δ*rck2* pCM161 and *S. cerevisiae* Δ*rck2* pCM161:*RCK2* in 15% glucose. Mean + SD (n = 3).
