# Supplementary figures and images for: Expression of RCK2 MAPKAP (MAPK-activated protein kinase) rescues yeast cells sensitivity to osmotic stress
Source: Microb Cell Fact. 2015 Jun 12;14:85. doi: 10.1186/s12934-015-0276-7 (PMC4464721; doi:10.1186/s12934-015-0276-7)

## Slide 1
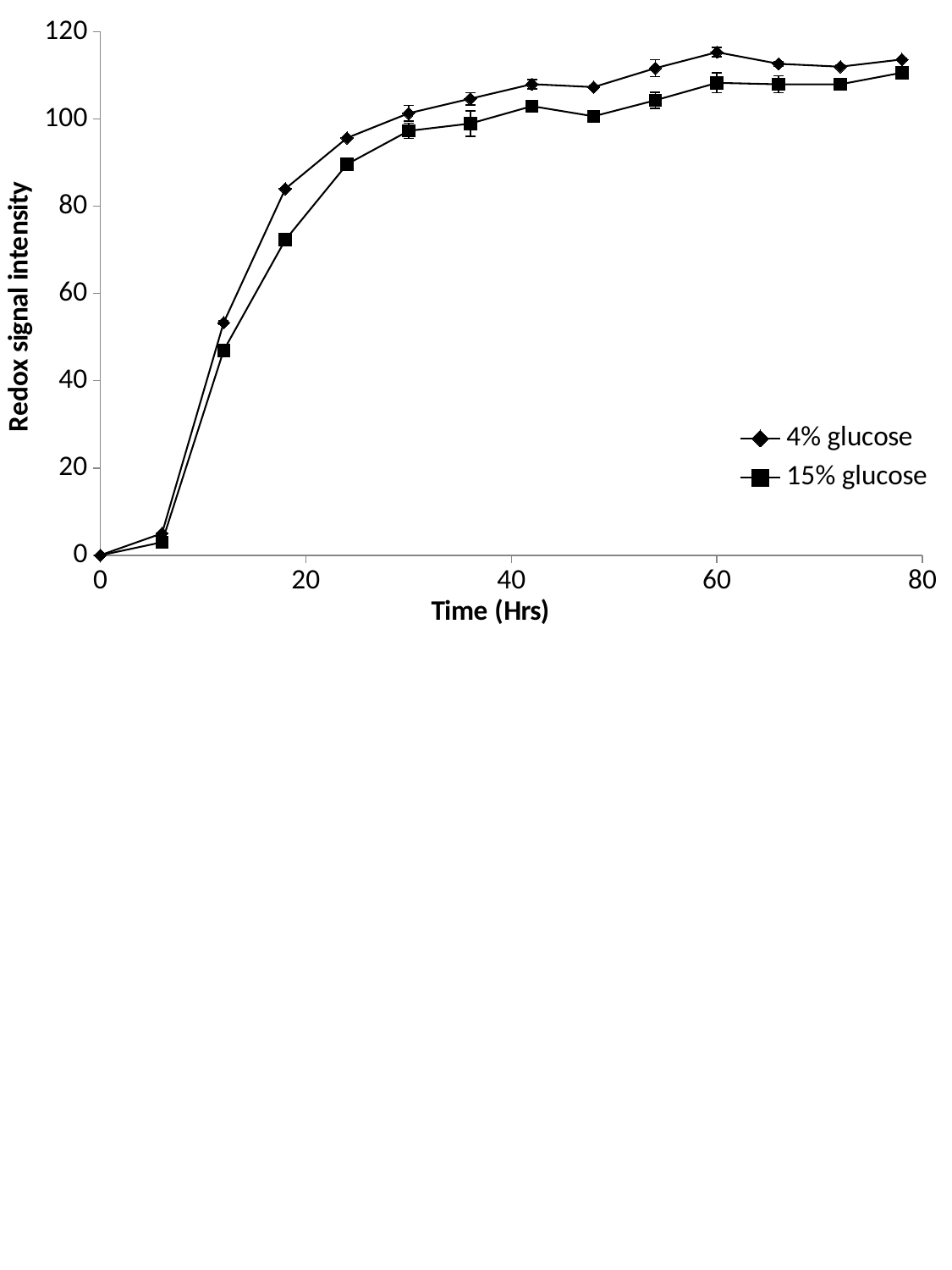

### Chart
| Category | 4% glucose | 15% glucose |
|---|---|---|

Supplement: Supplementary file 1 — Additional file 1: Figure S1. Phenotypic microarray analysis for S. cerevisiae Δrck2(pCM161:RCK2) under 4 and 15% glucose. Mean + SD (n = 3). [file 12934_2015_276_MOESM1_ESM.pptx]

## Slide 1
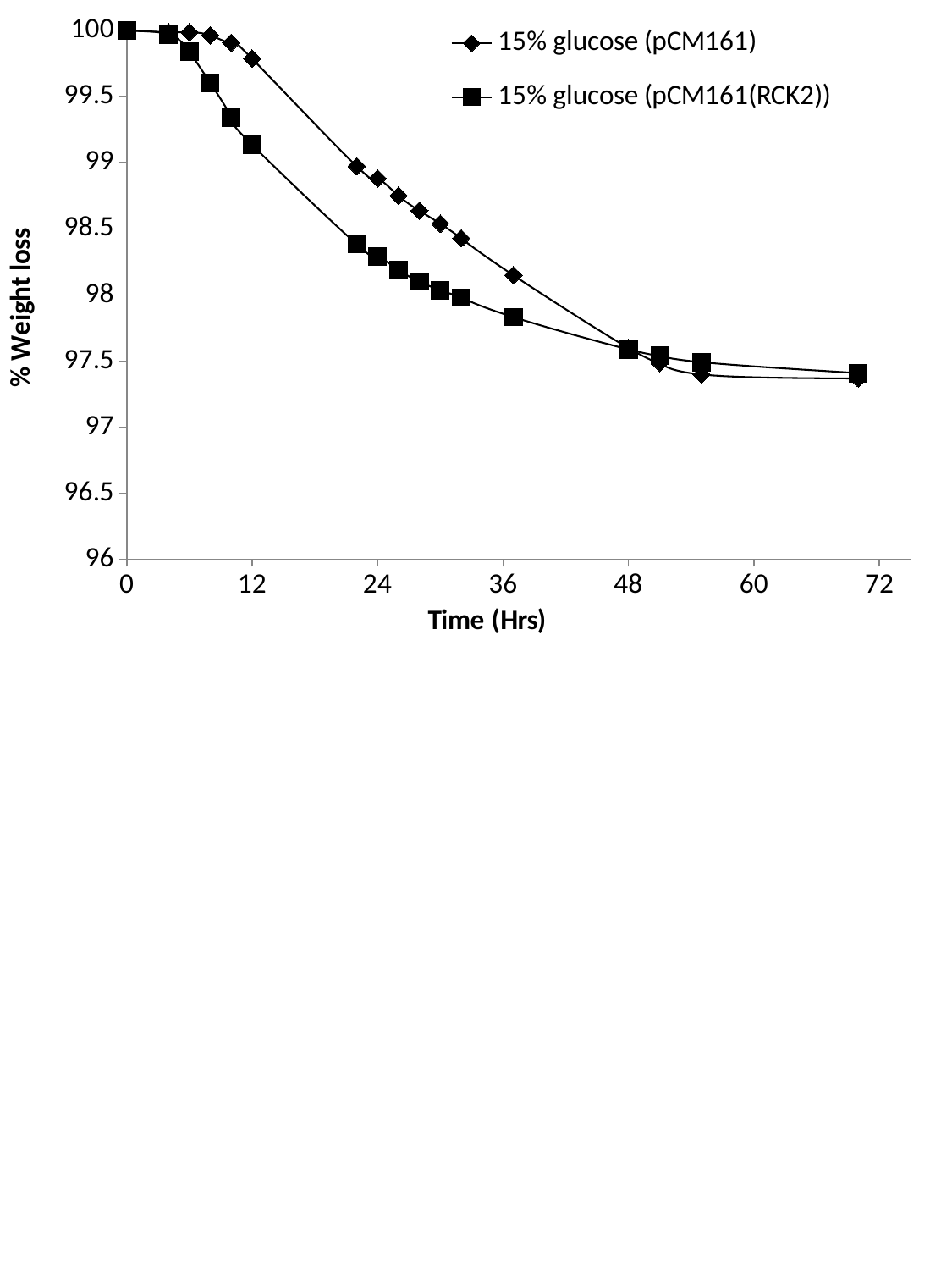

### Chart
| Category | 15% glucose (pCM161) | 15% glucose (pCM161(RCK2)) |
|---|---|---|

Supplement: Supplementary file 2 — Additional file 2: Figure S2. Fermentation profiles for S. cerevisiae Δrck2 pCM161 or S. cerevisiae Δrck2 pCM161:RCK2 under osmotic stress (A) performance of S. cerevisiae Δrck2 pCM161 and S. cerevisiae Δrck2 pCM161:RCK2 in 15% glucose. Mean + SD (n = 3). [file 12934_2015_276_MOESM2_ESM.pptx]
